# Point-of-sale cigarette marketing and smoking-induced deprivation in smokers: results from a population-based survey

**DOI:** 10.1186/s12889-016-2992-2

**Published:** 2016-04-28

**Authors:** Mohammad Siahpush, Raees A. Shaikh, Regina Robbins, Melissa Tibbits, Asia Sikora Kessler, Ghada Soliman, Molly McCarthy, Gopal K. Singh

**Affiliations:** University of Nebraska Medical Center, 984365 Nebraska Medical Center, Omaha, NE 68198-4365 USA; University of Nebraska Omaha, 6001 Dodge St, Omaha, NE 68182 USA; The Center for Global Health and Health Policy, Global Health and Education Projects, P O Box 234, Riverdale, MD 20738 USA

**Keywords:** Point-of-sale tobacco marketing, Smoking-induced deprivation, Cravings to smoke, Urge to buy cigarettes, Unplanned purchase of cigarettes

## Abstract

**Background:**

Strict restrictions on outdoor cigarette marketing have resulted in increasing concentration of cigarette marketing at the point-of-sale (POS). The association between POS cigarette marketing and smoking-induced deprivation (SID) has never been studied. The aim of this study was to examine this association and how it is mediated by cravings to smoke, urges to buy cigarettes, and unplanned purchases of cigarettes.

**Methods:**

Data from a telephone survey of 939 smokers were collected in Omaha, Nebraska. POS cigarette marketing was measured by asking respondents three questions about noticing pack displays, advertisements, and promotions such as cigarette price discounts within their respective neighborhoods. SID was measured with the following question: “In the last six months, has there been a time when the money you spent on cigarettes resulted in not having enough money for household essentials such as food? [yes/no]” We used structural equation modeling to examine the study aim.

**Results:**

There was overwhelming evidence for an association between higher levels of POS cigarette marketing and a higher probability of SID (*p* < 0.001). This association was partly mediated by cravings to smoke, urges to buy cigarettes, and unplanned purchases of cigarettes during a visit to a neighborhood store (*p* < 0.001).

**Conclusion:**

Given that POS cigarette marketing is associated with a higher probability of experiencing SID, policies that ban POS cigarette marketing might help some smokers afford essentials household items such as food more easily and thus have better standards of living.

## Background

As a result of the 1998 Master Settlement Agreement in the United States, a legal settlement that eliminated most forms of outdoor cigarette advertising such as on billboards, cigarette marketing has increasingly concentrated at the point of sale (POS) [1–3]. In 2011, about 89 % of the $8.4 billion of tobacco industry expenditures for cigarette marketing was made at the POS [4] in the following three areas: cigarette pack displays, advertisements, and price incentives to consumers [2–4].

POS cigarette marketing can act as a cue to smoke and promote cravings to smoke, urges to buy cigarettes, and impulse or unplanned purchases of cigarettes [5–14]. In an experimental study of 1216 current smokers and recent quitters, Kim et al. reported that exposure to an enclosed (invisible) display compared to an open display of cigarette packs in a virtual store resulted in a lower level of self-rated craving [5]. In a different experimental study of 63 smokers, Carter et al. found that self-rated craving to smoke was higher following exposure to a photo of eight cigarette packs than exposure to a neutral photo with no cigarette imagery [6]. Observational studies also indicate an association between exposure to pack displays and cravings to smoke. In a qualitative study, Hoek et al. conducted semi-structured in-depth interviews with 20 participants who had attempted to quit smoking in the previous six months [7]. Many participants indicated that seeing cigarette displays reminded them of smoking and promoted not only cravings but also impulse purchases of cigarettes. Similarly, in a cross-sectional study of 526 current smokers, Wakefield et al. found that the frequency of noticing cigarette displays was positively related to the probability of getting an urge to buy cigarettes and making an impulse purchase of cigarettes [8]. Similar findings were reported by Carter et al. who conducted intercept interviews with 206 smokers who were observed purchasing cigarettes from retail outlets and found that POS displays were associated with four times as many unplanned purchases as purchases that were planned [9]. About 22 % of the participants in Carter et al.’s study reported that they did not plan to purchase cigarettes before entering the store and 20 % indicated that cigarette pack displays encouraged them to purchase cigarettes in that instance. Finally, two other observational studies using the same sample of 999 smokers reported that POS displays and advertisements were associate with more frequent cravings to smoke [14] and that POS marketing (a summated scale consisting of items about exposure to POS displays, advertisement, and promotions) was associated with more frequent urges to buy and impulse purchases of cigarettes [13]. In the field of public health, the phrases “unplanned purchase” and “impulse purchase” have been used interchangeably. However, marketing literature makes a clear distinction between these two phrases. Based on this literature, while an unplanned purchase refers to a shopping decision made without any advanced planning, an impulse purchase refers to a shopping decision that in addition to being unplanned, involves a sudden, strong, and often irresistible urge to purchase [15, 16].

An area of research that has not previously been explored is the extent to which POS marketing and its immediate consequences such as stimulating cravings to smoke, urges to buy cigarettes, and unplanned purchases of cigarettes, can contribute to the deleterious effects of smoking on the standards of living of smokers. Research shows that spending money on cigarettes and smoking can diminish financial and material well-being [17–22]. In a cross-sectional study of 6,892 households, Siahpush et al. found that spending money on cigarettes was associated with an increased probability of experiencing financial stress (e.g. going without meals or not being able to pay rent due to shortage of money) and that among households with a smoker, spending more on tobacco was associated with a higher probability of financial stress [18]. In a different study of 5887 smokers, Siahpush et al. directly measured “smoking-induced deprivation” (SID) by asking respondents whether there was an instance in recent times where spending money on cigarettes resulted in not having enough money for necessities of life such as food [21] and found that those who spent more money on cigarettes were more likely to experience SID. This finding was replicated in a different cross-sectional study of 2,410 smokers [17].

**Fig. 1:**
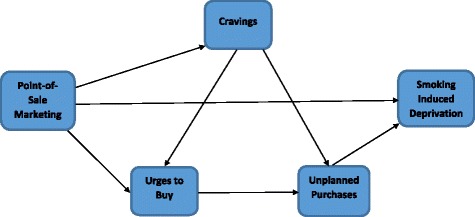
Hypothesized path model linking POS cigarette marketing to SID

While the association of POS cigarette marketing with cravings to smoke, urges to buy cigarettes, and unplanned purchases of cigarettes, on the one hand, and the association of smoking behavior with smoking-induced deprivation (SID), on the other hand, have been examined in previous literature, there are no studies that link these concepts together to investigate the relationship between POS cigarette marketing and SID. Based on the studies described above, we posited a conceptual framework depicted in Fig. 1. This model indicates that POS cigarette marketing can lead to cravings to smoke and urges to buy cigarettes during a visit to a store, which in turn can lead to unplanned purchases of cigarettes. Unplanned purchases of cigarettes can thus result in SID. We have also explored a direct effect of POS cigarette marketing on SID. Our aim was to use structural equation modelling to examine the relationship between POS cigarette marketing and SID and investigate how this relationship is mediated by cravings to smoke, urges to buy cigarettes, and unplanned purchases of cigarettes in a cross-sectional, population-based sample of smokers in Omaha, Nebraska.

## Methods

### Sample

A total of 999 adult respondents were recruited in Omaha, Nebraska, using random digit dialing (47.2 % with a response rate of 22.4 %) and placement of local advertisements (52.8 %) in media such as the major daily newspaper and Craigslist, in 2014. All data were collected using telephone interviews that took an average of 20 min. Those included in the study spoke English, were 18 years of age or older, were current smokers meaning that they had smoked more than 100 cigarettes in their life, [23] and smoked five or more cigarettes a day at the time of recruitment. We excluded very light smokers, i.e. current smokers who smoked less than 5 cigarettes a day, because they appear to be notably different from other smokers in relation to important smoking-related factors such as tobacco dependence, cravings to smoke before or after smoking cessation, [24] likelihood to make a quit attempt, post-cessation withdrawal symptoms, [25] and smoking motives [26]. Those who responded “never” to the following question were excluded from the study: “How often do you visit the stores in the neighborhood where you live? By stores, we mean such places as convenience stores, gas stations, grocery stores, supermarkets, drug stores, liquor stores, and tobacco stores.” Response options were 1 = never, 2 = sometimes, 3 = frequently, and 4 = always. The University of Nebraska Medical Center Institutional Review Board provided ethics approval for the study. Informed consent was obtained from each participant verbally as the data collection was done through telephone interviews.

While the study sample was not a random sample, its socio-demographic distribution was similar to the subsample of smokers in the center city of Nebraska Metropolitan Statistical Areas in the Behavioral Risk Factor Surveillance System (BRFSS) [27]. For example, the gender distribution in our sample and BRFSS were identical. The mean age was 47.8 years in our sample and 53 years in BRFSS. The percentage of Whites was 71.7 in our sample and 86.1 in BRFSS. The percentage of respondents with a high school diploma or a lower level of education was 49.9 in our sample and 46.3 in BRFSS. The median income was $22,500 in our sample and $30,000 in BRFSS.

### Measurement

#### Latent variables

We measured exposure to POS marketing with the following three survey items, which are adapted from previous studies: [8, 28] “When you are in a store in your neighborhood, how often do you notice tobacco ads?”; “When you are in a store in your neighborhood, how often do you notice tobacco promotions such as special prices, multi-pack discounts, or free gift with purchase of cigarettes?”; and “When you are in a store in your neighborhood, how often do you notice cigarette pack displays?” Possible responses to each question were: 1 = never, 2 = rarely, 3 = sometimes, 4 = often, 5 = always. Before asking these questions, respondents were told that in the study “store” refers to convenience store, gas station, grocery store, supermarket, drug store, liquor store, tobacco store, etc. where tobacco products are sold.

We measured cravings to smoke with the following three survey items: “When you are in a store in your neighborhood that sells tobacco products, how often do you (1) feel a craving for a cigarette? (2) feel like nothing would be better than smoking a cigarette? (3) feel like all you want is a cigarette?”. The response options were: 1 = never, 2 = rarely, 3 = sometimes, 4 = often, 5 = always [29–32].

#### Observed variables

SID was measured with the following question: “In the last six months, has there been a time when the money you spent on cigarettes resulted in not having enough money for household essentials such as food?” [17, 21, 33].

We measured urges to buy cigarettes and unplanned purchases of cigarettes using the following two questions, respectively, which were adapted from previous studies: [8, 28]: “When you are in a store in your neighborhood, how often do you get an urge to buy cigarettes?”; and “When you are in a store in your neighborhood to shop for something other than cigarettes, how often do you decide to buy cigarettes?” Possible response options were: 1 = never, 2 = rarely, 3 = sometimes, 4 = often, 5 = always.

We included the following control variables in the analyses: Heaviness of Smoking Index (HSI), which is an indicator of nicotine dependence, [34, 35] gender, age in years, race/ethnicity, household income, education, frequency of visiting stores, and method of recruitment (random digit dialing versus other). Race was categorized as non-Hispanic White, non-Hispanic Black, Hispanic, and other. Education was categorized based on highest grade or year of school completed as follows: less than high school, high school graduate, some college, and college graduate and higher.

### Statistical analysis

We used Stata v. 13 for descriptive statistics [36] and Mplus [37] to perform structural equation modeling (SEM) to address the aim of the study. SEM is a multivariate technique that estimates parameters in structural equations in order to simultaneously examine complex relationships between several independent and dependent variables and estimate direct, indirect, and total effects. SEM can accommodate latent variables with multiple indicators to isolate and remove measurement error and enhance predictive power [38]. Observations that had a missing value on any of the study variables, which constituted 6 % of the original sample, were omitted from the analysis. The analysis sample size was 939. We examined the association of missingness with sociodemographic factors and the study outcome. There was very little evidence that missingness was associated with gender (*n* = 999; *p* = 0.669), race/ethnicity (*n* = 996; *p* = 0.057), education (*n* = 998; *p* = 0.769), or SID (*n* = 998; *p* = 0.054). However, there was evidence that the mean age of the individuals in the analysis was lower than that of those not included in the analysis (*n* = 994; *p* = 0.003).

We performed SEM in two stages [39]. First, using maximum likelihood parameter estimation with standard errors and mean- and variance-adjusted (MLMV) chi-square test statistics for continuous data, [37] we estimated a measurement model involving POS cigarette marketing and cravings to smoke, each with three indicators. Next, using probit regression and robust weighted least square parameter estimation with standard errors and mean- and variance-adjusted (WLSMV) chi-square test statistic for binary outcome, [37] we estimated a structural model representing both latent (POS marketing and cravings to smoke) and observed variables of interest (urge to buy cigarettes, unplanned purchase of cigarettes, and SID) in the study and specifying the pathways connecting these variables [37]. Where appropriate, we used the modification index to estimate additional parameters to enhance the fit of a model. We used the DIFFTEST procedure, which provides a χ^2^ difference test, to compare nested models. We included all of the control variables as exogenous observed variables in structural equations. If the *p*-value for the effect of a control variable was greater or equal to 0.05 in any of the equations, it was removed from that equation. We used comparative fit index (CFI), Tucker-Lewis index (TLI), and root mean square error of approximation (RMSEA) to assess the fit of the models. A model was considered to have a good fit with the observed data if the following were true: CFI > = 0.95, TLI > =0.95, and RMSEA < = 0.05 [40, 41]. Standardized coefficients representing the direct effects were presented in the structural equation diagram. Standardized coefficients are expressed in terms of standard deviation units and as such provide a measures of the strength of association, are used as an effect size index, and allow a comparison of different effects within the same model [42]. Estimates of total and indirect effects (Muthén B: Applications of causally defined direct and indirect effects in mediation analysis using SEM in Mplus, unpublished working paper) of POS on SID were also provided. All reported coefficients are standardized regression coefficients or*βs.* It should be noted that, as is customary in describing the results of SEM, we use the word “effect” to describe the association between variables rather than to ascribe a causal nature to the observed pattern of associations.

## Results

### Sample characteristics

Table 1 shows the characteristics of the sample. Eleven and a half percent of respondents reported having experienced SID in the past six months. The mean of exposure to POS cigarette marketing was 3.1 (SD: 1.5) for displays, 3.1 (SD: 1.6) for advertisements, and 2.8 (SD: 1.3) for promotions. The means of feeling a craving for a cigarette, that nothing would be better than a cigarette, and that all that is wanted is a cigarette were 2.8 (SD: 1.3), 2.8 (SD: 1.2), and 2.9 (SD: 1.2), respectively. The mean of urge to buy cigarettes and unplanned purchase of cigarettes were 3 (SD: 1.3) and 2.6 (SD: 1.2), respectively. The mean level of HSI was 3.3 (SD: 0.9). The percentage of men was 57.2. Mean age was 47.5 (SD: 14.2). Respondents who were non-Hispanic White, non-Hispanic Black, and Hispanic comprised 65.8 %, 24.2 % and 3.1 % of the sample, respectively. Mean income was $31,000 (SD: $23,230) and 49.9 % of the sample had finished high school or had a lower level of education. The percentage of respondents who visited the stores in their neighborhoods sometimes, frequently, or always, was 11.5, 36.8, and 51.6, respectively. About 45.3 % of the analysis sample were recruited by random digit dialing.

**Table 1:** Sample characteristics (*n* = 939)

Variables	% or mean (range, standard deviation)
SID	
Yes	11.5
No	88.5
POS marketing	
Displays	3.11 (1.48)
Ads	3.13 (1.55)
Promotions	2.79 (1.33)
Cravings	
Craving for a cigarette	2.75 (1.34)
Nothing better than a cigarette	2.76 (1.24)
All you want is a cigarette	2.94 (1.15)
Urges to buy cigarettes	3.02 (1.31)
Unplanned purchases	2.58 (1.2)
HSI	3.27 (0.92)
Sex	
Male	57.19
Female	42.81
Age	47.5 (14.23)
Race/ethnicity	
Non-Hispanic White	65.81
Non-Hispanic Black	24.17
Hispanic	3.09
Other	6.92
Income ($1000)	31.08 (23.23)
Education	
Less than high school	10.12
High school graduate	39.83
Some college	36.95
Frequency of visits to stores	
Sometimes	11.50
Frequently	36.85
Always	51.65
Method of recruitment	
Random digit dialing	45.26
Other	54.74

### Measurement model

The measurement model with POS cigarette marketing and cravings to smoke as latent variables provided good fit to the data (CFI = 0.98; TLI = 0.96; RMSEA = 0.06). An examination of the modification indices indicated that adding a parameter for a correlation between the error terms of two of the indicators of cravings to smoke, namely “feel like nothing would be better than smoking a cigarette” and “ feel like all you want is a cigarette”, would further enhance the fit of the model. Data provided support for adding this parameter (χ^2^ for difference: 22.36, 1df, *p* < 0.001) and the indices of fit improved (CFI = 1; TLI = 0.99; RMSEA = 0.03). In this revised measurement model, the *p*-values for all the loadings (standardized coefficients) were smaller than 0.001.

### Structural model

**Fig. 2:**
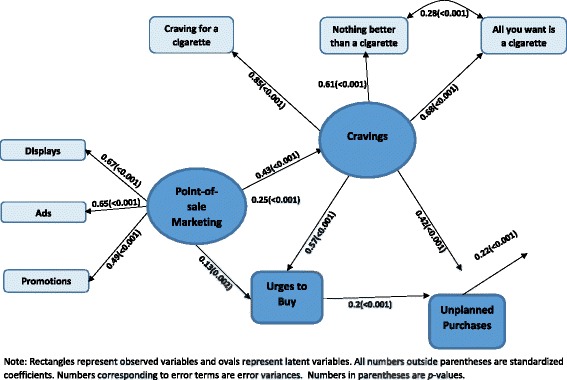
Structural equation model for the relationship between POS cigarette marketing and smoking-induced deprivation (SID)

The path diagram depicted in Fig. 1, which represents the conceptual framework of the study, was further refined to include the measurement model, as shown in Fig. 2. In this figure, ovals represent latent variables and rectangles represent observed variables. For simplicity of presentation, control variables are not included in the diagram. The model provided a good fit to the data (CFI = 0.97; TLI = 0.96; RMSEA = 0.02). As indicated by the *p*-values associated with each path, there was overwhelming evidence that POS cigarette marketing was associated with cravings to smoke, urges to buy cigarettes, and SID; cravings to smoke was associated with urges to buy cigarettes and unplanned purchases of cigarettes; urges to buy cigarettes were associated with unplanned purchases of cigarettes; and unplanned purchases of cigarettes were associated with SID. All of these relationships were positive, showing that higher levels of POS marketing and unplanned purchases of cigarettes were associated with a higher probability of SID. Examining the magnitude of the unstandardized coefficient in Fig. 2 indicates the following: the direct effect of cravings to smoke on urges to buy cigarettes was notably greater than the direct effect of POS marketing on urges to buy cigarettes; the direct effect of cravings to smoke on unplanned purchases of cigarettes was notably larger than the direct effect of urges to buy cigarettes on unplanned purchases of cigarettes; and the direct effect of POS marketing on SID was very similar to the direct effect of unplanned purchases of cigarettes on SID.

Table 2 shows the effect of control variables on the endogenous variables. None of the control variables were associated with urges to buy cigarettes. Females, older respondents, and Whites (compared to other races or ethnic groups) were exposed to lower levels of POS marketing. Having a lower HSI level, being male, being older, and having a higher level of income or education were associated with a lower frequency of cravings to smoke. Compared to other race/ethnicities, Whites reported a lower frequency of unplanned purchases of cigarettes. Respondents with a higher level of HSI, and those who were non-Hispanic White (compared to other race/ethnicities) had a higher probability of SID.

**Table 2:** Adjusted standardized coefficients and p-values (in parentheses) for the effect of control variables on POS marketing, cravings to smoke, unplanned purchases of cigarettes, and SID (*n* = 939)

Control variables	POS marketing	Cravings to smoke	Unplanned purchases	SID
HSI	--	0.14 (<0.001)	--	0.11(0.033)
Sex				
Male	0.13 (<0.001)	−0.11 (0.001)	--	--
Female	0	0	--	--
Age	−0.024 (<0.001)	−0.09(0.018)	--	--
Race/ethnicity				
Non-Hispanic White	0	--	0	0
Non-Hispanic Black	0.11 (0.008)	--	0.09 (0.005)	−0.14 (0.016)
Hispanic	0.08 (0.022)	--	0.04 (0.126)	−0.04 (0.393)
Other	0.03 (0.459)	--	0.02 (0.418)	0.05 (0.238)
Income ($1000)	0.13 (0.002)	-.10 (0.015)	--	−0.42 (<0.001)
Education				
Less than high school	--	0	--	--
High school graduate	--	−0.12 (0.034)	--	--
Some college	--	−0.19 (0.001)	--	--
Frequency of visits to stores				
Sometimes	0	0	--	--
Frequently	0.16 (0.016)	0.13 (0.026)	--	--
Always	0.28 (<0.00)	0.14 (0.013)	--	--
Method of recruitment				
Random digit dialing	−0.15 (<0.001)	−0.08 (0.033)	−0.09 (0.003)	--
Other	0	0	0	--

Table 3 shows the total, direct, and indirect effects of POS marketing on SID. The direct effect accounted for 81.6 % and the total indirect effect accounted for 18.4 % of the total effect of POS cigarette marketing on SID. While the data supported all three indirect effects, most of the total indirect effect was via cravings to smoke and unplanned purchases of cigarettes.

**Table 3:** Decomposition of the effect of POS cigarette marketing on SID

	*β*	*p*-value
Total effect	0.31	<0.001
Direct effect	0.25	<0.001
Total indirect	0.06	<0.001
Indirect effects		
via cravings, urges to buy, unplanned purchases	0.01	0.001
via cravings, unplanned purchases	0.04	<0.001
via urges to buy, unplanned purchases	0.01	0.03

## Discussion

In this study, we used SEM to investigate the previously-unexplored relationship between exposure to POS cigarette marketing and SID. We found strong evidence that POS cigarette marketing is associated with SID and that part of this association was mediated by cravings to smoke, urges to buy cigarettes, and unplanned purchases of cigarettes during a visit to a store.

The experience of SID and other forms of financial deprivation that result from smoking [43–46] are important not only because they indicate compromised standards of living, but also because they can lead to further unfavorable smoking behaviors and outcomes. For example, smokers who experience SID are less likely to attempt to quit smoking and those who do try to quit are more likely to relapse [43]. Similarly, smokers who experience financial stress are less likely to quit and ex-smokers who experience financial stress are more likely to relapse [45, 46]. In short, the relationship between financial deprivation and smoking is reciprocal and smokers are often caught in a vicious cycle of experiencing financial deprivation because of smoking and not being able to quit because of the stress associated with financial deprivation [44]. Our finding that POS cigarette marketing is associated with SID indicates that POS cigarette marketing can further exacerbate this vicious cycle.

Due to the cross-sectional nature of this study, its findings cannot be used to establish causality. While we controlled for several important predictors of SID in the analyses and used structural equation modelling with suggested directions of causality, our analysis was correlational and in the absence of a controlled experimental study, we are unable to make strong conclusions about causality.

A weakness of the measurement of many of our central constructs was that it relied on respondents’ recollection of past events and behaviors. In particular, we measured SID by asking respondents to recall whether “in the past six months” they spent money on cigarettes that “resulted in not having enough money for household essentials”. In addition to the issue of the difficulty recalling past events, the wording of our question might have led some respondents to confirm that smoking would result in financial deprivation.

## Conclusion

With the passage of the Family Smoking Prevention and Tobacco Control Act in 2009, the Food and Drug Administration in the US gained the authority to regulate the marketing of tobacco products. Studies such as ours, suggesting that a reduction in cigarette marketing might improve the standards of living of smokers, can strengthen the evidence base needed by the FDA to ban all forms of POS cigarette marketing, as is the case in countries such as Australia, Canada, Croatia, Finland, Iceland, New Zealand, Norway, Russia, Thailand, and the UK.

### Ethical approval statement

Institutional Review Board at University of Nebraska Medical Center provided the ethical approval for this study.

### Availability of data and materials

De-identified, limited data will be shared by the lead author upon request.

## Abbreviations

BRFSS: behavioral risk factor surveillance system
CFI: comparative fit index
HSI: heaviness of smoking index
POS: point of sale
RMSEA: root mean square error of approximation
SID: smoking induced deprivation
SEM: structural equation modelling
SD: standard deviation
TLI: tucker-lewis index
