# Erratum to: ‘Point-of-sale cigarette marketing and smoking-induced deprivation in smokers: results from a population-based survey’

**DOI:** 10.1186/s12889-016-3178-7

**Published:** 2016-06-08

**Authors:** Mohammad Siahpush, Raees A. Shaikh, Regina Robbins, Melissa Tibbits, Asia Sikora Kessler, Ghada Soliman, Molly McCarthy, Gopal K. Singh

**Affiliations:** University of Nebraska Medical Center, 984365 Nebraska Medical Center, Omaha, Nebraska USA; University of Nebraska Omaha, Omaha, Nebraska USA; The Center for Global Health and Health Policy, Global Health and Education Projects, P O Box 234, Riverdale, MD 20738 USA

Unfortunately, the original version of this article [1] contained an error. The presentation of Fig. 2 was incorrect. The correct version is given below.

**Fig. 2:**
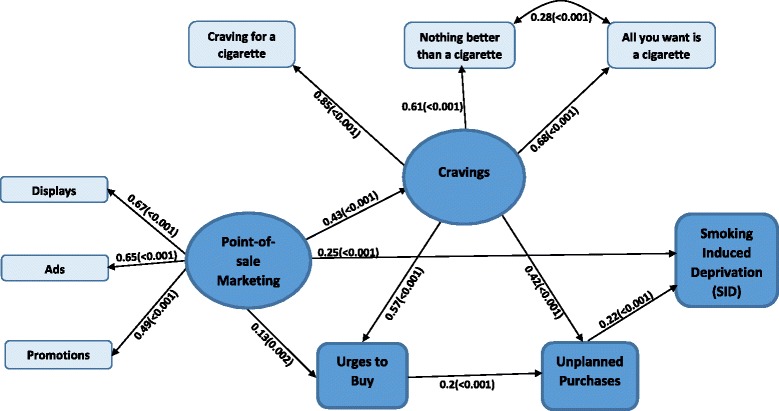
Structural equation model for the relationship between POS cigarette marketing and smoking-induced deprivation (SID)
